# RiboProfiling: a Bioconductor package for standard Ribo-seq pipeline processing

**DOI:** 10.12688/f1000research.8964.1

**Published:** 2016-06-09

**Authors:** Alexandra Popa, Kevin Lebrigand, Agnes Paquet, Nicolas Nottet, Karine Robbe-Sermesant, Rainer Waldmann, Pascal Barbry

**Affiliations:** 1 Institut de Pharmacologie Mol´eculaire et Cellulaire, University Nice Sophia Antipolis and CNRS, Sophia- Antipolis, 06560, France

**Keywords:** ribosome profiling, genomics, ribosome footprints

## Abstract

The ribosome profiling technique (Ribo-seq) allows the selective sequencing of translated RNA regions. Recently, the analysis of genomic sequences associated to Ribo-seq reads has been widely employed to assess their coding potential. These analyses led to the identification of differentially translated transcripts under different experimental conditions, and/or ribosome pausing on codon motifs. In the context of the ever-growing need for tools analyzing Ribo-seq reads, we have developed ‘RiboProfiling’, a new Bioconductor open-source package. ‘RiboProfiling’ provides a full pipeline to cover all key steps for the analysis of ribosome footprints. This pipeline has been implemented in a single R workflow. The package takes an alignment (BAM) file as input and performs ribosome footprint quantification at a transcript level. It also identifies footprint accumulation on particular amino acids or multi amino-acids motifs. Report summary graphs and data quantification are generated automatically. The package facilitates quality assessment and quantification of Ribo-seq experiments. Its implementation in Bioconductor enables the modeling and statistical analysis of its output through the vast choice of packages available in R. This article illustrates how to identify codon-motifs accumulating ribosome footprints, based on data from *Escherichia coli*.

## Introduction

Ribosome profiling (Ribo-seq) is a recently developed high throughput sequencing technique ( Ingolia *et al.*, 2009) that allows the identification of RNA fragments resistant to RNAse digestion. Fragments mainly correspond to coding sequences protected against RNAse digestion by translating ribosomes. Ribo-seq data have been widely used to assess the translational status of open reading frames (ORFs) ( Bazzini *et al.*, 2014; Fields *et al.*, 2015; Ingolia *et al.*, 2009; Popa *et al.*, 2016), and to identify ORFs differentially translated between experimental conditions ( Schafer *et al.*, 2015).

Ribo-seq bioinformatics analyses comprise the selection of reads consistent with ribosome footprints, a recalibration of the read start or end (5’ or 3’ extremity of the read depending on the RNA digestion step) to the peptidyl site (P-site) position of the ribosome, and quantification of the reads on specific features of interest (i.e. transcript, codons, multi-codons motifs). Several tools for processing ribosome profiling data have previously been proposed. RiboTools’ ( Legendre *et al.*, 2015) and ‘RUST’ ( O’Connor *et al.*, 2015)) were developed in python, ‘riboSeqR’ ( Hardcastle, 2014) corresponds to an R package. Each of the above software integrates some, but not all, of the functions necessary for a standard Ribo-seq workflow from reads to quality assessment, recalibration and quantification. A previous effort to group the different approaches for Ribo-seq analyses has been developed with the Galaxy instance RiboGalaxy ( Michel *et al.*, 2016).

These tools have been developed to answer specific questions related to ribosome occupancy: normalization of Ribo-seq reads (‘RUST’), detection and characterization of reading frame usage (‘riboSeqR’), or irregular translational behavior such as translational ambiguities (Ribo-seq footprints in different phases) and stop-codon read through (‘RiboTools’). However, prior to any advanced Ribo-seq data processing, it is necessary to have standard pipelines for quality assessment of the experiments and specific ribosome footprint assignment to sequences.

We implemented the features for a standard Ribo-seq workflow in a pipeline entitled ‘RiboProfiling’. The pipeline takes an alignment file (BAM) as input, performs identification of the read offset, generates transcript and (multi-) codon coverage quantification data, and performs statistical analyses as well as graphical representations. Our pipeline is, to our knowledge, the most complete integration of a ribosome profiling standard analysis pipeline in an unique R framework. This includes the crucial step prior to quantifying ribosome footprints that consists in identifying the offset between Ribo-seq reads and the P-site of the ribosome. We have given special attention to this step as it is essential to correctly associate ribosome footprints with codon resolution. Depending on the RNA digestion protocol, the assignment of the ribosome must be made specifically either to the 5’, 3’ or the center of the read. To our knowledge, there is only one implementation for the determination of ribosome offset that was published so far. It was proposed in ‘riboSeqR’ as a metagene plot and the determination of the offset was only possible from the 5’ read end and for a particular read length. Our package offers several options, to compute the offset and recalibrate reads based either on the 5’ or the 3’ read ends. RiboProfiling also enables the graphical representation of ribosome density around the Translation Start Site (TSS) for multiple read lengths. This option allows to perform analysis for a single read length or on the merge of several read lengths. It enables to group all lengths sharing a same and unique offset value. The R/Bioconductor implementation provides an easy-to-use comprehensive set of functions that requires a minimal knowledge in R programming. The package contains a function entitled ‘riboSeqFromBAM’ that treats multiple Ribo-seq BAM files in parallel. The automated workflow generates report summary graphs and data quantification.

We illustrate the main features of the package using a Ribo-seq control sample from murine ES cells (GSM1655059), taken from a recently published ribosome profiling study using translation inhibitors ( Popa *et al.*, 2016). We then detail the analysis of ribosome accumulation on certain codons and tri-peptide motifs on a public dataset in *Escherichia coli* (GSE64488) ( Woolstenhulme *et al.*, 2015). The script for performing all these analyses is publicly available.

## Methods

### Requirements

The ‘RiboProfiling’ package, v.1.2.0 can be used with R 3.3.0 and Bioconductor version 3.3. The script needs a minimum memory limit in R of 3 Gb when analyzing tripeptide motifs. The package starts from alignment BAM files, from either Ribo-seq or RNA-seq experiments. We have validated BAM files from bowtie/tophat, Hisat2, STAR, and Lifescope (Solid), both single- or paired-end (for RNA-seq reads). Reads from rRNA, tRNA, and PCR duplicates (if unique molecular identifiers are available) should be removed from the BAM files before starting the analysis (see package vignette for details).

### RiboProfiling package

**Figure 1.**
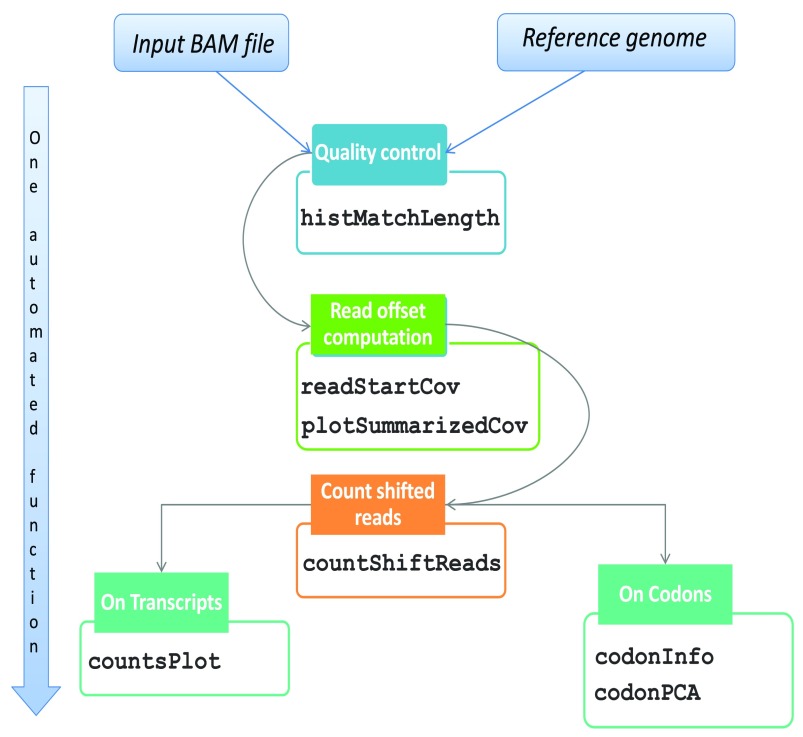
Workflow of ‘RiboProfiling’ Ribo-seq analysis from BAM to quantification on genomic features and codon motifs. A second particularity in the handling of Ribo-seq data comes from the shift existing between the extremities of the read (i.e. 5’ or 3’) and the P-site position of the ribosome. Our package allows the identification of an offset from the 5’ end of the read, but also from the 3’ end. The function ‘readStartCov’ computes the read frequency distribution centered on the translation start site (TSS) of the most expressed protein coding transcripts (by default the 3% most expressed). Based on this frequency distribution, the ‘plotSummarizedCov’ function enables the visual quantification of the offset between the reads and the ribosome P-site ( Figure 2.B). In our Ribo-seq example, the 5’ read end is shifted 13 bp from the TSS. The innovation of this feature consists in the visualization of read lengths independently and as a summary figure.

**Figure 2.**
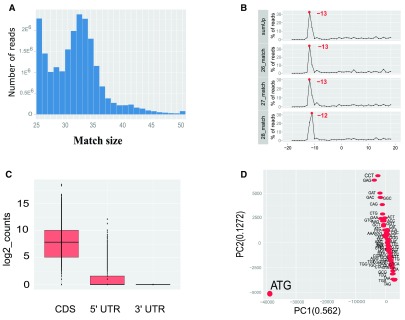
Graphical output of ‘RiboProfiling’ package on the GSM1655059 dataset. **A**, Histogram of read length distribution with ‘histMatchLength’ function. **B**, Frequency of reads around the TSS for reads sizes 26 to 28; this graph points to an offset of 13 bp between the read 5’ end and the ribosome P-site. **C**, Boxplots of Ribo-seq read coverage on the CDS, 5’ UTR and 3’ UTR regions of protein coding genes. **D**. PCA analysis of Ribo-seq coverage on codons.

All analyses can be performed either through a call to a function called ‘riboSeqFromBAM’, or through a step by step approach. Figure 1 describes the workflow of the package starting from BAM files with reads mapped to the genome of interest. The first step in processing Ribo-seq reads is to select only those with match lengths compatible with standard ribosome footprints. The function ‘histMatchLength’ allows the visual inspection of read match sizes distribution across the BAM file ( Figure 2.A), which should be enriched in reads of sizes between 20 to 40 nucleotides ( Ingolia *et al.*, 2009; Popa *et al.*, 2016).

**Figure 3.**
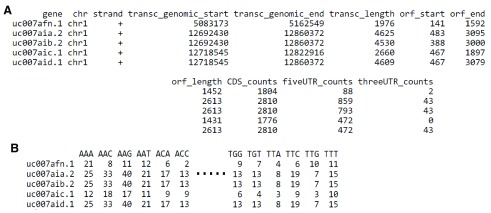
Output tables of ‘RiboProfiling’ package on the GSM1655059 dataset. **A**, Quantification of Ribo-seq read coverage on the CDS, 5’ UTR and 3’ UTR regions of protein coding genes. **B**. Quantification of Ribo-seq coverage on codons.

When computed, the offset can be applied on all reads based on the transcript referential with the function ‘countShiftReads’ and coverage on three different sequence features: 5’-UTR, coding sequences (CDS), and 3’-UTR ( Figure 3.A). In Figure 2.C we observe that the majority of reads accumulate on the CDS of protein coding sequences and are practically lacking in the 3’ non-coding UTR regions. Ribosome footprints are also detectable in the 5’ UTR regions of protein coding genes, suggesting either the presence of coding upstream ORFs ( Popa *et al.*, 2016) or possible confounding information from missing annotations.

Finally, our package provides quantification of ribosome footprints at codon resolution ( Figure 3.B). A PCA analysis of codon occupancy can be performed and several graphical functions are implemented. In Figure 2.D we employed the ‘codonInfo’ and ‘codonPCA’ functions to analyze the codons accumulating ribosome footprints. As expected, the codon ATG is the most discriminant codon in the PCA analyses, since ribosome accumulation peaks are observed at the start codon of coding regions. Detailed descriptions with examples of the pipeline from BAM files to Ribo-seq reads quantification and processing are available in the vignette of our package: https://www.bioconductor.org/packages/release/bioc/vignettes/RiboProfiling/inst/doc/RiboProfiling.pdf.

## Analysis of ribosome stalling on sequence motifs

‘RiboProfiling’ can also be useful for analyzing ribosome occupancy on multi-codons motifs. Codons accumulating ribosome footprints are indicative of slowed ribosome progression (stalling) during the translation elongation process. The ‘RiboProfiling’ package offers several features for quantifying footprint accumulation on sequence motifs (ranging from one to three consecutive codons), performs principal component analyses, and allows graphical representation of those data.

**Figure 4.**
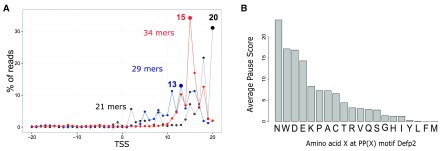
**A**, Sample ΔEfp2: percentage of read 3’ end coverage 20 nucleotides around the TSS. Three match sizes are represented: 21, 29 and 34 mers. **B**, Barplot of the average ribosome occupancy on PP(X) motifs in the ΔEfp2 sample, where X is any of the 20 possible amino acids.

To illustrate how ‘RiboProfiling’ can be used to explore the influence of sequence motifs (in this case tri-amino-acid sequences) on ribosome pausing, we analyzed an *Escherichia coli* Ribo-seq dataset ( Woolstenhulme *et al.*, 2015). We downloaded, filtered and mapped the reads of an efp-knockout sample (ΔEfp2, GSE64488), the elongation factor EFP being essential for the translation of polyproline motifs. Uniquely mapped reads from the resulting BAM file were analyzed with our ‘RiboProfiling’ package. After quality assessment of the reads size distribution, we quantified the offset between the 3’ end of the reads and the TSS for different alignment match sizes ( Figure 4.A). We can clearly observe the 15 nucleotides offset that was reported by the authors for reads with alignment sizes >= 30 nucleotides. Smaller match lengths exhibited either a strong variation in the distribution of reads around the TSS (i.e. the 29 mers), or a different offset (i.e. offset of 20 for 21mers) ( Figure 4.A). We selected the reads with alignment match sizes between 30 and 40 nucleotides and quantified codon coverage by positioning the ribosome P-site 15 nucleotides upstream of the read 3’ extremity.

An important stalling has been reported after the incorporation of two consecutive prolines (Pro-Pro) in the peptide chain. This stalling is highly dependent on the nature of the codon that follows the aminoacyl-tRNA reacting at the A-site ( Woolstenhulme *et al.*, 2015). Following the article’s analysis of stalling at PP(X) (Proline – Proline – 3rd codon) motifs, we used the ‘countShiftReads’ and ‘codonInfo’ functions to quantify the ribosome footprints on these motifs, in the ΔEfp2 sample. We then computed the pause score for all 20 possible PP(X) combinations in each ORF with more than 20 ribosome footprints, independently:

$$PauseScore_{PPX|ORF}=\frac{\frac{Reads_{PPX|ORF}}{Reads_{ORF}}}{\frac{Nbr_{PPX|ORF}}{Length_{ORF}}}$$

where, *Reads _PPX|ORF_* is the ribosome density of motif PPX in a given ORF; *Reads _ORF_* is the ribosome density on the ORF; *Nbr _PPX|ORF_* is the number of time a given PPX motif is present in the ORF; *Length _ORF_* is the total length of the ORF. We averaged the ribosome occupancy for each PP(X) motif on all ORFs. Figure 4.B shows a strong stalling in sample ΔEfp2 when the ribosome encounters PPN, PPW, PPD, in agreement with their previous identification as pause sites *in Escherichia coli* ( Woolstenhulme *et al.*, 2015). A step by step R script implementing this entire analysis is provided at:

http://genomique.info/data/public/RiboProfiling/scriptWoolstenhulme_Defp2.R.

## Summary

Our ‘RiboProfiling’ Bioconductor package offers a collection of tools for Ribo-seq data analysis. It provides an unique, straightforward R implementation of a ribosome profiling pipeline from BAM, to P-site calibration, quantification of reads on sequence features, and codon coverage. The packages’ graphical features offer quality assessment and result representation across the analyses. Following the overview of Ribo-seq experiments with ’RiboProfiling, the output tables can then be easily integrated into more specialized dowstream analyses, either using more specialisez riboseq tools such as (XXX, YYY) or directly within R.

We highlighted here the features of our package in characterizing ribosome stalling at sequence motifs along ORFs based on an example dataset from Woolstenhulme *et al.* ( Woolstenhulme *et al.*, 2015). The workflow we propose for the analysis of ribosome occupancy on codon motifs using the ‘RiboProfiling’ package will most surely prove an useful asset in the context of recent ribosome profiling applications such as the detection of tumor sensitivity to differential amino acid depletion ( Loayza-Puch *et al.*, 2016).

## Software availability

The package is built in R (>=3.3.0) and freely available from Bioconductor website

1. https://www.bioconductor.org/packages/release/bioc/html/RiboProfiling.html
2. We provide an associated-script to the analyses in this paper at http://genomique.info/data/public/RiboProfiling/scriptWoolstenhulme_Defp2.R
Zenodo: scriptWoolstenhulme_Defp2.R, doi: 10.5281/zenodo.54567, ( Popa A, 2016)
3. GPL-3 licence
