# Supplementary material for: RiboProfiling: a Bioconductor package for standard Ribo-seq pipeline processing
Source: F1000Res. 2016 Jun 9;5:1309. [Version 1] doi: 10.12688/f1000research.8964.1 (PMC4918025; doi:10.12688/f1000research.8964.1)
Supplement: Peer review history

# Peer review: Referee response for version 1

**DOI:** 10.5256/f1000research.9644.r14283

**Published:** 2016-07-05

**Authors:** Ghislain Bidaut

**Affiliations:** 1 Integrative Bioinformatics, Inserm, Aix-Marseille Université, CNRS and Institut Paoli-Calmettes, Centre de Recherche en Cancérologie de Marseille, Marseille, France

I started my review by trying to reproduce the vignette example. I also made sure that all documentation was present in the reference package PDF, which was the case.

 As stated in the paper, the Riboprofiling package has to be installed under R version 3.3 minimum. I installed the latest version at the date of review, which was 3.3.1+ BioC 3.3.

 I started by the installation of the Riboprofiling package, which posed no problem.

 I am not proficient with existing pipelines, and did not verify if Riboprofiling is more complete than them.

 However, I found that an R implementation is nice since most users will be familiar with its installation, organisation and mode of use and won’t have to find other tools elsewhere.

 I have a few remarks on the installation and on the vignette example:

 I had to install the Bioconductor Rsamtools which was not installed alongside Riboprofiling, while other dependencies were installed.

 After running the covData <- riboSeqFromBAM(listInputBam, genomeName="hg19 »), I obtained the following warnings. Not sure if they impact the final result, but I nevertheless report them.

 Warning messages:

 1: In riboSeqFromBAM(listInputBam, genomeName = "hg19") :

   paramScanBAM parameter is not a ScanBamParam object. Set to default NULL value!

 2: In doTryCatch(return(expr), name, parentenv, handler) :

   [knet_seek] SEEK_END is not supported for HTTP. Offset is unchanged.

 3: In doTryCatch(return(expr), name, parentenv, handler) :

   [bam_header_read] EOF marker is absent. The input is probably truncated.

 4: In countShiftReads(exonGRanges[names(cdsPosTransc)], cdsPosTransc,  :

   Param motifSize should be an integer! Accepted values 3, 6 or 9. Default value is 3.

 5: In doTryCatch(return(expr), name, parentenv, handler) :

   [knet_seek] SEEK_END is not supported for HTTP. Offset is unchanged.

 6: In doTryCatch(return(expr), name, parentenv, handler) :

   [bam_header_read] EOF marker is absent. The input is probably truncated.

 7: In countShiftReads(exonGRanges[names(cdsPosTransc)], cdsPosTransc,  :

   Param motifSize should be an integer! Accepted values 3, 6 or 9. Default value is 3.

 In the vignette page 5, I would add the statement

 library(GenomicAlignments)

 before the readGAlignments call in order to make the code easier to work..

 Vignete page 6. The instruction

 txdb <- TxDb.Hsapiens.UCSC.hg19.knownGene

 does not work. I do not have a TxDb.Hsapiens.UCSC.hg19.knownGene object in my session. I couldn’t go further in running the provided tutorial. Could you clarify on how to obtain that object ?

 Apart from the vignette, the paper itself is well written and can be followed simply through. An analysis case of ribosome stalling study is presented through a complete example and R code (analysis of Woolstenhulme et al dataset). The code works without problem until the invocation of library(BSgenome.Ecoli.NCBI.K12.MG1655). I couldn’t get this package on the Bioconductor Web Site, and some clarification might be helpful for this step.

 Minor remark:

 The authors missed a couple of references in the last paragraph:  (...) either using more specialisez riboseq tools such as (XXX, YYY) or directly within R.

I have read this submission. I believe that I have an appropriate level of expertise to confirm that it is of an acceptable scientific standard.

# Peer review: Referee response for version 1

**DOI:** 10.5256/f1000research.9644.r14282

**Published:** 2016-06-21

**Authors:** Audrey M Michel

**Affiliations:** 1 School of Biochemistry and Cell Biology, University College Cork, Cork, Ireland

RiboProfiling allows a number of ribo-seq specific analyses to be carried out using a single Bioconductor package in R and is supported by instructions in the corresponding reference and 'package' manuals. The automatic assignment of the P-site from either the 5' or 3' end of the ribosome footprint read is a useful feature. In addition the tool allows the quantification of the distribution of footprints across coding regions (CDS), 5' leader and 3' trailer regions at the metagene and individual gene transcript level. The authors use the reported stalling events in the Woolstenhulme *et al.,* 2015 ^1^ ribo-seq data to illustrate how RiboProfiling can be used to determine the ribosome occupancy on multiple codon motifs.

 Overall, the RiboProfiling package allows some ribo-seq specific analyses to be automatically carried out using the RiboProfiling 'quick command' or by using the step-by-step commands. However I would argue that RiboProfiling is not more complete than existing tools. There is no assessment of triplet periodicity for example. The quality assessment carried out by RiboProfilig is the distribution of footprint read lengths (Figure 1). While determining the read lengths in a ribo-seq dataset is a useful assessment, it is only one determinant of the quality of ribo-seq data. The RUST tool (O'Connor *et al.* 2015 ^2^) as mentioned by the authors carries out normalisation of the data, but also allows for the quality of the ribo-seq and mRNA-seq data to be determined in terms of sequencing biases.

 Nevertheless, RiboProfiling adds to the repertoire of publicly available ribo-seq tools which is useful to the community.

**Questions:**

  

1. In the manuscript the abbreviation TSS is used for Translation Start Site. However, on page 6 of the RiboProfiling manual “aroundPromoter: returns the genomic positions flanking the **transcript start site** (TSS) for the x% (3% default value) best expressed CDSs”. Should this be Translation start site?
2. It is not clear what the authors mean by tools such as (XXX, YYY) ' in the following sentence in the Summary section 'Following the overview of Ribo-seq experiments with ‘RiboProfiling, the output tables can then be easily integrated into more specialized dowstream analyses, either using more specialisez riboseq tools such as (XXX, YYY) or directly within R.' (There are also some typo errors in this sentence (dowstream; specialisez).
3. While following the instructions in the RiboProfiling manual, there were several operations for which I was missing the required library (e.g. library(Rsamtools), library(GenomicAlignments), library(TxDb.Hsapiens.UCSC.hg19.knownGene), etc).  While it might be obvious which additional libraries are required after running the command, it may help users if they were specified in the manual along with library(RiboProfiling).
4. I ran the 'quick start' riboSeqFromBAM function on one of our own datasets and four plots were generated fine. However, the 'list of per ORF per codon coverage' was not output. Should this have been generated as stated in the manual?
5. What read lengths are considered in the RiboProfiling 'quick command' approach for the offset determination? On our own dataset the sumUp offset plot was generated showing an offset of -13 (which corresponds to our own estimation). However, this plot did not show the individual read lengths. Are read lengths sharing the same offset value used?
6. All of the step-by-step commands in the user manual ran fine on our own dataset and the corresponding plots were generated. However, there was an error when I tried to run the accompanying scriptWoolstenhulme_Defp2.R to generate the barplot in Figure 4B of the average ribosome occupancy on PP(X) motifs in the E.coli dataset (see further). So unfortunately I was unable to verify this part of the manuscript.
7. According to the manual, “consecutive motifs of 9 nucleotides (3 consecutive codons) overlap on 6 nucleotides. The Ribo-seq coverage is reported as the coverage on the 2nd codon in the motif considered as being in the P-site.” Is the average pause score in Figure 4B for ribosome occupancy of the second codon (P) or the last codon (X) or across the three codons?
8. The scriptWoolstenhulme_Defp2.R is hard-coded for the PPX motif and E.coli. I did not get to check if it can be easily adapted for other codon motifs in other datasets.

Below I provide a sub-set output of the scriptWoolstenhulme_Defp2.R with the error. If the sessionInfo() details can help troubleshoot the error, I can send it to the authors separately so as not clog up this report.

 ….

 Import genomic features from the file as a GRanges object ... trying URL 'http://genomique.info/data/public/RiboProfiling/Escherichia_coli_str_k_12_substr_mg1655_no_rRNA_no_tRNA.gtf'

 Content type 'unknown' length 2766800 bytes (2.6 MB)

 ==================================================

 downloaded 2.6 MB

 OK

 Prepare the 'metadata' data frame ... OK

 Make the TxDb object ... OK

 [knet_seek] SEEK_END is not supported for HTTP. Offset is unchanged.

 [bam_header_read] EOF marker is absent. The input is probably truncated.

 [knet_seek] SEEK_END is not supported for HTTP. Offset is unchanged.

 [bam_header_read] EOF marker is absent. The input is probably truncated.

 Error in if (length(ixLengthPb)/length(testLength) >= 0.7) { : 

   missing value where TRUE/FALSE needed 

 Calls: readStartCov -> readStartCov1Aln -> normRange 

 Execution halted

I have read this submission. I believe that I have an appropriate level of expertise to confirm that it is of an acceptable scientific standard.

## References

[1] WoolstenhulmeCJGuydoshNRGreenRBuskirkAR: High-precision analysis of translational pausing by ribosome profiling in bacteria lacking EFP. *Cell Rep*.2015;11(1) : 10.1016/j.celrep.2015.03.014 13-21 10.1016/j.celrep.2015.03.014 25843707PMC4835038

[2] O'ConnorP: Surveying the relative impact of mRNA features on local ribosome profiling read density in 28 datasets. *bioRxiv*.2015; 10.1101/018762 10.1101/018762 PMC505944527698342

# Peer review: Referee response for version 1

**DOI:** 10.5256/f1000research.9644.r14285

**Published:** 2016-06-20

**Authors:** Olivier Namy, Pierre Bertin

**Affiliations:** 1 Institute for Integrative Biology of the Cell (I2BC), University of Paris-Sud, Orsay, France; 2 Université Paris-Sud, Orsay, France

This article represents a useful package to analyze ribosome profiling data. We found that several important features of the Ribosome Profiling data processing are well implemented in this Bioconductor Package. Actually, the Kmer sizes distribution, the P-site location, the features (CDS, 5UTR, 3UTR) coverage distribution and the codon coverage are important and significant to explain Ribosome Profiling data and this package generates a great summary of these. However this package is not as complete as suggested by the authors. Indeed an important point is the periodicity of footprints that is not addressed here. Moreover it could be interesting to add the ability to detect which reading phase is indeed read. This should be feasible since the P-site is well defined.

 I include below a few comments for the authors.

- The algorithm of the readStartCov function is not sufficiently described. The only one sentence found, does not give enough information to understand how it works. For instance, "the read frequency distribution centered on the translation start site": "Centered" needs more explanation. This is important, because using our own datasets we were not able to obtain any convincing results about the position of the P-site. The lack of information prevented us from understanding why.
- In the Package Manual, when the aroundPromoter function is defined, the TSS is described as the "Transcript Start Site", which does not match with the "Translational Start Site" found in the article.
- The pipeline requires an annotation file with a specific format (TxDb object). Users have to be aware of that if they want to use their own genome.

We have read this submission. We believe that we have an appropriate level of expertise to confirm that it is of an acceptable scientific standard.
